# Hydrothermal synthesis of NiWO_4_ crystals for high performance non-enzymatic glucose biosensors

**DOI:** 10.1038/srep24128

**Published:** 2016-04-18

**Authors:** Sivakumar Mani, Veeramani Vediyappan, Shen-Ming Chen, Rajesh Madhu, Veerakumar Pitchaimani, Jia-Yaw Chang, Shang-Bin Liu

**Affiliations:** 1 Department of Chemical Engineering and Biotechnology, National Taipei University of Technology, Taipei 10608, Taiwan; 2 Institute of Atomic and Molecular Sciences, Academia Sinica, Taipei 10617, Taiwan; 3 Department of Chemical Engineering, National Taiwan University of Science and Technology, Taipei 10607, Taiwan; 4 Department of Chemistry, National Taiwan Normal University, Taipei 11677, Taiwan

## Abstract

A facile hydrothermal route for the synthesis of ordered NiWO_4_ nanocrystals, which show promising applications as high performance non-enzymatic glucose sensor is reported. The NiWO_4_-modified electrodes showed excellent sensitivity (269.6 μA mM^−1 ^cm^−2^) and low detection limit (0.18 μM) for detection of glucose with desirable selectivity, stability, and tolerance to interference, rendering their prospective applications as cost-effective, enzyme-free glucose sensors.

It is well-known that an abnormal level of glucose in human blood may cause metabolic diseases such as diabetes mellitus, endocrine metabolic disorder, eye defect, and cardiovascular diseases. As such, the detection of glucose plays a vital role in various applications such as biotechnology, bio-processing, clinical research, and food industry^1^,^2^,^3^. Likewise, research and development on high-performance glucose biosensors with desirable sensitivity and selectivity is an imperative task. Among various methods available for electrochemical detections of glucose, they are commonly catalogued by either enzymatic or non-enzymatic biosensors. The more conventional glucose biosensors were mostly fabricated based on glucose oxidase (GOx) enzymes, which catalyzes the oxidation of glucose (Glu) to hydrogen peroxide and D-glucono-δ-lactone (GDL), also known as gluconolactone. However, although these enzyme based biosensors exhibit highly sensitive performance, their universal application is limited and hampered by drawbacks such as sophisticated immobilization and stabilization protocol of the enzyme and activity hindrances due to pH, temperature, humidity, and toxic chemicals and so on, leading to poor stability and reproducibility of the sensors^4^,^5^,^6^. To unravel these problems, R&D of enzyme-free electrochemical sensors have becoming a highly desirable alternative owing to their characteristics such as easy fabrication, high sensitivity, fast response, low detection limit, and cost-effectiveness^7^,^8^.

In view of the urgent demand in developing new materials for applications in the high-performance glucose biosensors, various studies of non-enzymatic glucose sensors based on metals (e.g., Pt, Au)^9^,^10^ and alloys^11^ have been reported. However, these costy metals showed only fair antitoxic capability, operational stability, and selectivity. Alternatively, electrochemical biosensors based on transition-metal oxides (such as NiO, Co_3_O_4_, CuO, and TiO_2_) and hydroxides were found to exhibit desirable catalytic activity with high sensitivity in alkaline medium^7^,^12^,^13^,^14^,^15^. Along the same line, many literature reports have been made available regarding to applications of mixed transition-metal oxides/sulfides in a wide variety of different fields such as energy storage, catalysis, and biosensing^16^,^17^,^18^,^19^,^20^. Among them, R&D of mixed metal tungstates (MWO_4_; where M = transition-metals such as Ni, Co etc.) have drawn considerable attentions owing to their extraordinary physicochemical properties and practical applications as electrode materials in various areas, e.g., photo anodes, supercapacitors, memory devices, humidity sensors, and so on^21^,^22^,^23^,^24^,^25^,^26^. For example, Niu *et al*. demonstrated^23^ that nanostructured NiWO_4_ materials prepared by a simple co-precipitation method showed notable enhancement in electrical conductivity in the order of 10^−7^ to 10^−3^ S cm^−1^ at different temperatures compared to pure NiO (typically, *ca*. 10^−13^ S cm^−1^) due to the presence of various valance states provoked by the incorporated W atoms^27^.

We report herein, the preparation of octahedron-like nickel tungstate (NiWO_4_) crystals by using a facile hydrothermal method and their performances as enzyme-free glucose sensors, as illustrated in Fig. 1. To the best of our knowledge, this is the first report on application of NiWO_4_ microcrystals for non-enzymatic glucose sensors. It is found that the NiWO_4_-modified glassy carbon electrodes (GCE) exhibit good electrocatalytic activity toward the oxidation of glucose. Moreover, the biosensors so fabricated show superior analytical parameters, such as wide linear range, excellent sensitivity, and lower detection limit, desirable for electrochemical detection of glucose.

## Results and Discussion

**Figure 2:**
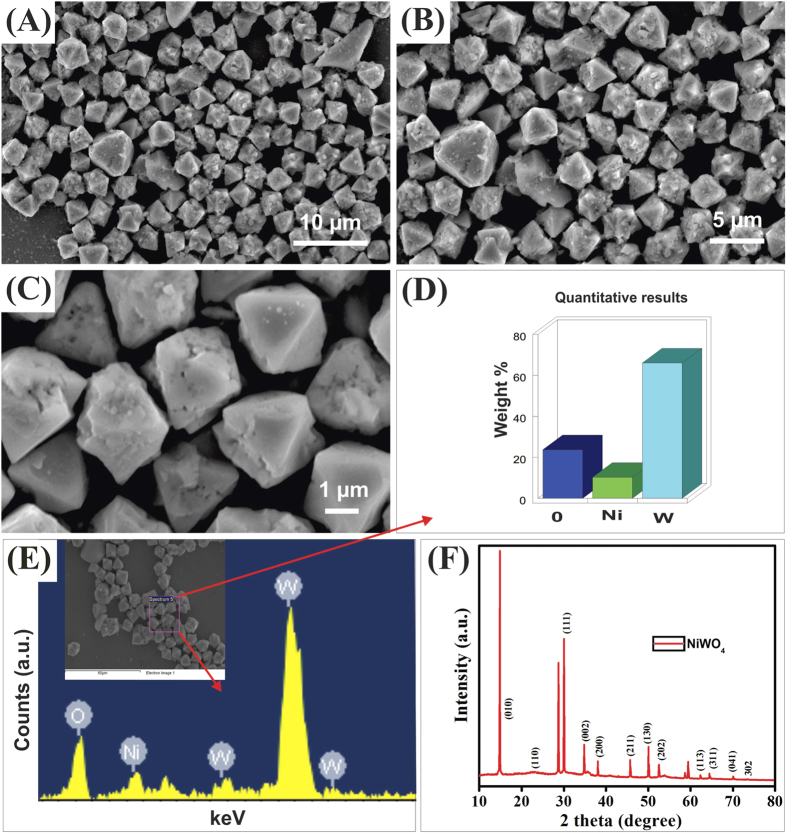
(**A**–**C**) SEM images, (**D**,**E**) EDX results, and (**F**) XRD profile of the as-synthesized NiWO_4_ microcrystals.

Figure 2A–C displays the scanning electron microscopy (SEM) images of the as-synthesized NiWO_4_ material, which revealed the octahedron-like morphology with an average crystalline size of *ca*. 2.0 ± 0.1 μm. Further analysis by energy dispersive X-ray (EDX; Fig. 2E) confirmed the presences of anticipated elements, namely oxygen (O), nickel (Ni), and tungsten (W) with a concentration of ca. 25, 5 and 70 wt%, respectively (Fig. 2D). Moreover, the X-ray diffraction pattern (XRD) profile of the as-prepared NiWO_4_ in Fig. 2F shows diffraction peaks accountable for the (010), (110), (011), (111), (002), (200) planes, and so on, which match with that of crystalline NiWO_4_ (JCPDS file no. 15-0755)^28^,^29^.

**Figure 3:**
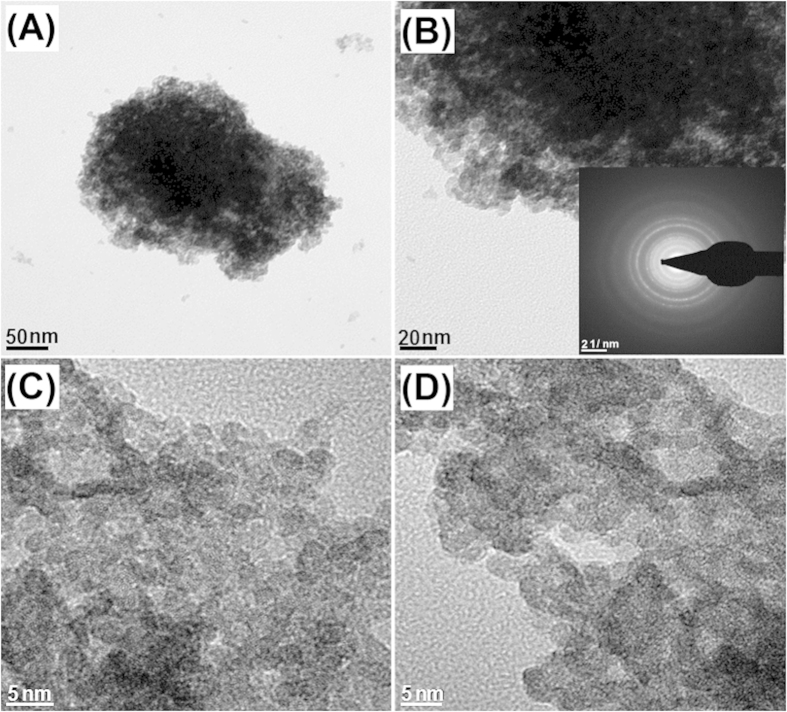
TEM images of the as-synthesized NiWO_4_ microcrystals. Inset in (**B**) shows the corresponding SAED pattern.

**Figure 4:**
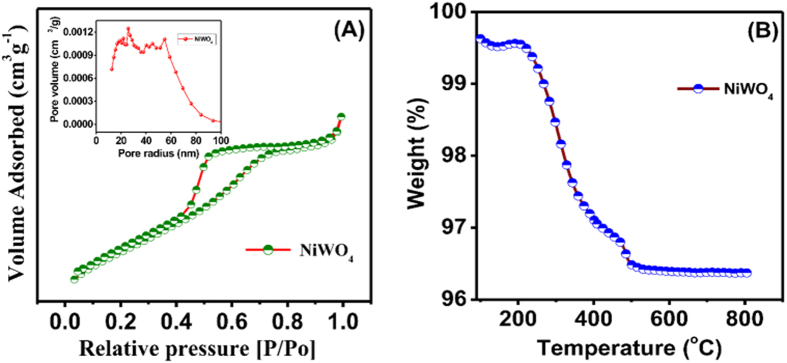
(**A**) N_2_ adsorption/desorption isotherms and (**B**) TGA curve of the as-synthesized NiWO_4_. Inset in (**A**) shows the pore size distribution profile.

The transmission electron microscope (TEM) images of the as-prepared NiWO_4_ material (Fig. 3) reveal crystals composing of aggregated nanoparticles with an average particle size of *ca*. 10–20 nm. The selected area electron diffraction (SAED) pattern in Fig. 3B (inset) clearly show the anticipated bright spots with rings, indicating the presence of particles with high crystallinity,in good agreement with the XRD data (Fig. 2F). The textural properties of the as-synthesized NiWO_4_ crystals were studied by nitrogen (N_2_) adsorption/desorption isotherms at 77 K, as shown in Fig. 4A. The NiWO_4_ crystals exhibited the type-IV isotherm curve with a sharp capillary condensation steps at a relative pressure (P/P_0_) range of 0.43–0.63, which revealed characteristics of a mesostructured materials. Accordingly, the corresponding Brunauer-Emmet-Teller (BET) surface area, total pore volume, and Barrett-Joyner-Halenda (BJH) pore size determined by the adsorption branch of the isotherm was found to be 70.7 m^2 ^g^−1^, 0.07 cm^3 ^g^−1^, and 4.19 nm, respectively. Furthermore, the thermal stability of the NiWO_4_ material was assessed by means of thermogravimetric analysis (TGA), as shown in Fig. 4B. The weight-loss at temperatures less than 100 °C (ca. 0.25 wt%) is attributed to the desorption of water molecules, whereas, the notable weight-loss (ca. 3.2 wt%) in the temperature range of 220–480 °C may be attributed to the decomposition of small amount of grafted metal hydroxides. Whilst the marginal weight-loss beyond 480 °C is most likely due to the formation of intermediate compounds^30^.

The NiWO_4_-modified GCE was also found to possess superior electrochemical properties than that of bare GCE, as verified by electrochemical impedance spectroscopy (EIS). As shown in Fig. S1 of the Supplementary information (hereafter denoted as SI), the Nyquist plot observed for the NiWO_4_-modified GCE in 0.1 M KCl solution containing 5 mM [Fe(CN)_6_]^3−/4−^ electrolyte exhibits a much higher charge-transfer resistance (R_ct_) compared to that of the bare GCE. This is clearly indicated by the larger diameter of the semicircle in the Nyquist plot observed for the NiWO_4_-modified GCE. Nevertheless, the R_ct_ value so obtained for the NiWO_4_-modified GCE is still much lower than that of pure NiO^31^. Thus, it is indicative that the NiWO_4_-modified electrode possesses a higher electrical conductivity and elelctron transfer rate, hence, more suitable for the electrochemical detection of glucose.

**Figure 5:**
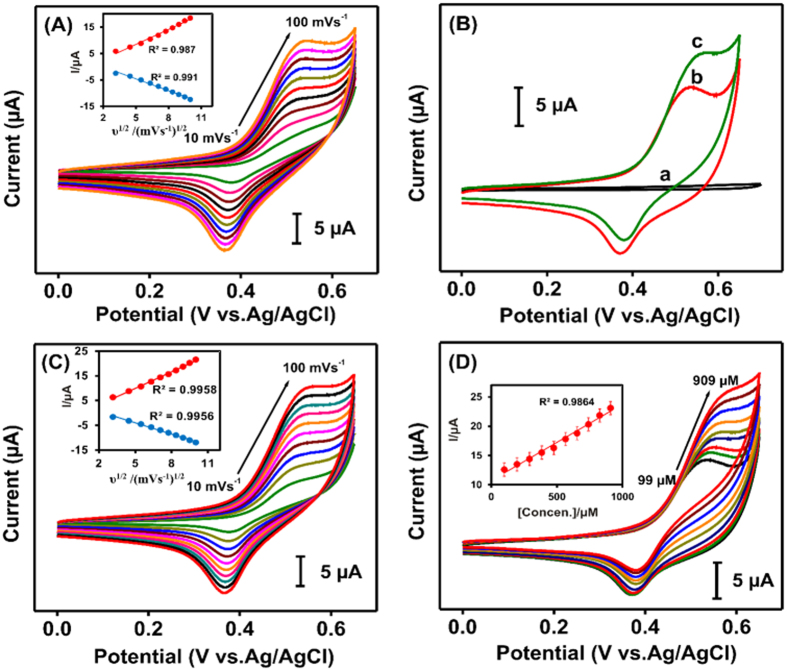
Electrochemical performances of NiWO_4_-modified electrodes. CV curves recorded (**A**) without and (**C**) with the presence of glucose (100 μM) in 0.1 M NaOH electrolyte solution at different scan rates (10–100 mV s^−1^). (**B**) Comparisons of CV curves obtained from (a) bare GCE, and NiWO_4_-modified GCEs (b) without and (c) with glucose (100 μM glucose) recorded with a scan rate of 50 mV s^−1^. (**D**) CV curves recorded under varied concentrations of glucose (99–909 μM). All insets show corresponding calibration plots.

Figure 5A displays the CV curves of the blank NiWO_4_-modified GCE recorded in 0.1 M NaOH electrolyte solution at different scan rates (10–100 mV s^−1^) while in the absence of glucose. Both anodic (*I*_pa_) and cathodic (*I*_pc_) redox peak currents as well as their peak-to-peak separation were found to increase linearly with increasing scan rate (see inset, Fig. 5A), indicating the occurance of a surface-controlled electrochemical process. By comparison, such redox peaks were invisible in bare GCE (curve a; Fig. 5B). In the absence of glucose, the neat NiWO_4_-modified GCE exhibited well-defined redox peak potentials (*E*_*pa*_ and *E*_*pc*_) of 0.51 and 0.37 V at a fixed scan rate of 50 mV s^−1^ (curve b; Fig. 5B), resembling that of the neat NiO-modified GCE^32^. In this context, while the existence of WO_4_^2−^ polyanions in the NiWO_4_ composite helps to promote a higher electrical conductivity, the presence of NiO should play the key role for the observed redox behavior^24^,^33^,^34^, which may be explained by the mechanism proposed by Wang *et al*.^15^:


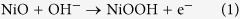


However, upon introducing glucose (100 μM) onto the NiWO_4_-modified GCE, a notable increase in *I*_pa_ along with a decrease in *I*_pc_ was observed (curve c; Fig. 5B), which may be ascribed due to formation of gluconolactone through oxisation of glucose by NiOOH^15^:


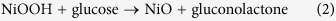


Interestingly, the glucose oxidation peak current (4.1 μA) observed for the NiWO_4_-modified GCE is higher than other Ni-based glucose sensors reported in literature (see Table S1; SI) even at such a low glucose concentration (100 μM). Again, this is attributed to the synergetic effect fast molecular diffusion, rapid electron transfer rate, and existence of active adsorption sites in the NiWO_4_ microcrystals. In addition, a linear correlation was also observed for both the oxidation (*I*_pa_) and the reduction (*I*_pc_) peak current vs scan rate (inset, Fig. 5C), respectively, while in the presence of glucose, revealing a surface-controlled process. Moreover, a linear correlation was also found between the oxidation peak current (*I*_pa_) and glucose concentration (inset, Fig. 5D).

**Figure 6:**
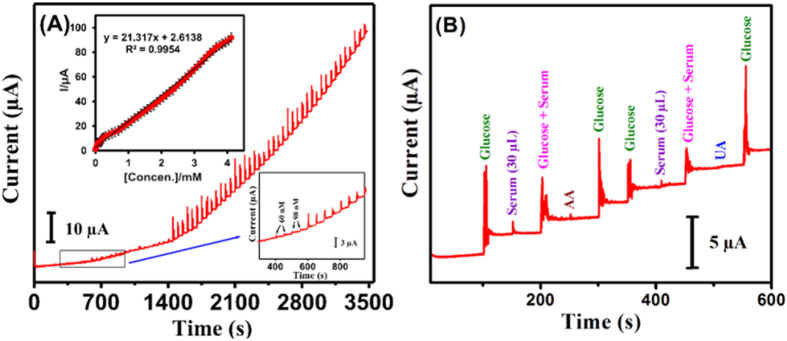
Amperometric response of NiWO_4_-modified GCE (**A**) under successive addition of glucose within the total concentration range from 0.006 μM to 4.1 mM; insets: (upper) corresponding calibration plot of peak current vs glucose concentration, (lower) blow-up response curve, and (**B**) obtained from anti-inference studies under the sequential influence of glucose and electroactive interferences (100 μM), viz. serum (30 μL), serum and glucose, AA, and UA. All measurements were conducted under the conditions: supporting electrolytes, 0.1 M NaOH aqueous solutions; rpm, 1200; applied potential, 0.55 V.

To evaluate the sensitivity and selectivity of the proposed glucose sensor, we performed amperometric *I-t* study using the NiWO_4_-modified GCE as the rotating disc electrode. Figure 6A shows the corresponding amperometric response during successive addition of glucose recorded in 0.1 M NaOH electrolyte solution at an applied potential of +0.55 V. Clearly, a linear correlation between the oxidation peak current and total glucose concentration (from 0.006 μM to 4.1 mM) may be inferred (inset, Fig. 6A). Accordingly, *ca*. lower detection limit (LOD) was 0.18 μM according to the formula LOD = 3 sb/S (where s_b_ is the standard deviation of the blank signal, and S is the sensitivity), the obtained sensitivity of the glucose sensor was derived to be 269.6 μA mM^−1 ^cm^−2^, surpassing most Ni-based composite GCEs reported in literatures (see Table S1; SI).

To assess the specificity of the enzyme-free glucose sensor for real-time applications, similar amperometric study was performed on NiWO_4_-modified GCE under sequential influence of 100 μM glucose and/or other electroactive interferences such as serum (30 μL), ascorbic acid (AA), and uric acid (UA), as shown in Fig. 6B. Obviously, the reported NiWO_4_-based biosensor is highly selectively for glucose detetion and insensitive towards other interfenence biomolecules. Moreover, the reported non-enzymatic glucose sensor is also insensitive to (glucose-free) serum, which contains a variety of proteins and other common interference molecules. By comparing the amperometric responses of the sensor in the presence of glucose with and without serum, it is clear that the reported NiWO_4_-modified electrode is indeed sensitive and selective for the detection of glucose. It has been reported that the zeta potential value of the CuWO_4_ is negative (−20 mV) in wide pH range 3–7 35. Thus, its surface is negatively charged in the pH range 3–7. In evidence, it has also been reported that SnWO_4_ nanoparticles possess similar negative zeta potential values (−20 mV) in the pH range 3–8, which increased significantly at higher pH values (−40 to −60 mV), revealing the negative surface charges on the SnWO_4_ nanoparticles greatly increase in alkaline media^36^. As glucose oxidation is carried out at the NiWO_4_ electrode in 0.1 M NaOH (pH 13), the surfaces of NiWO_4_ are expected to be negatively charged. As a result, the negatively charged surfaces of NiWO_4_ exhibit repelling effect that eliminates the negatively charged interferring species such as AA and UA, providing excellent anti-interference ability. As a result glucose molecules diffuse readily to the electrode surface, wherein the Ni (II/III) redox process efficiently mediates the glucose oxidation, providing greater selectivity.

The long-term stability of the reported glucose sensor was also tested by recording CV curves in the presence of 100 μM glucose under 0.1 M NaOH electrolyte solution for up to 50 consecutive cycles. It was found that the glucose sensor retained ca. 97.2% of its initial osidation peak potential value, revealing an excellent stability. Moreover, the reproducibility of the reported glucose sensor was also examined by performing CV studies of five independently prepared NiWO_4_-modified GCEs under similar conditons (100 μM glucose under 0.1 M NaOH electrolyte solution). The results so obtained revealed a good reproducibility with a relative standard deviation (RSD) of 2.7%.

In summary, NiWO_4_ microcrystals were synthesized by using a simple hydrothermal method and were employed for the first time as non-enzymatic glucose sensors. The NiWO_4_-modified electrodes exhibit not only ultra-high sensitivity and selectivity for detection of glucose even in the presence of bio-interferences such as serum, AA, and UA, but also show excellent low detection limit and detection in human blood serum samples. Interestingly, we achieved excellent analytical parameters such as lower detection limit, wide linear range, and good stability and reproducibility. Thus, these NiWO_4_-based electrodes, which show superior electrochemical performances surpassing other Ni-based electrodes, should have perspective applications as high-performance glucose sensors even in real samples.

## Experimental

### Materials

The NiWO_4_ materials were prepared by a hydrothermal method following the conventional procedures. Typically, *ca*. 6 mM of Na_2_WO_4_♦2H_2_O was add into a beaker containing 25 mL of deionized water. The mixture solution was sonicated for about 10 min before adding 6 mM of NiCl_2_♦6H_2_O solution in a dropwise manner under continuous stirring condition at room temperature. Subseqeuntly, the reaction mixture was placed in a 40 mL capacitive Teflon-lined stainless steel autoclave and treated at 180 °C for 5 h. Finally, the light green color precipitate was repeatedly washed with deionized water and ethanol several times and dried for overnight. The power sample was further calcined in air at 300 °C for 2 h to improve the crystallinity. All chemicals were obtained commercially and used as received without further purification.

### Characterization methods

The as-synthesized NiWO_4_ samples were characterized by a variety of physicochemical techniques. Their structure and morphology were monitored by field-emission scanningelectron microscope (FE-SEM; JEOL JSM-6700F) and field emission-transmission electron microscopy (FE-TEM; JEOL JEM-2100F). X-ray diffraction (XRD) studies were performed on a Rigaku, MiniFlex II instrument. N_2_ adsorption-desorption isotherms were measured on a Micromeritics ASAP 2020 apparatus. All electrochemical experiments were carried out on a CHI 611A electrochemical analyzer (CH instruments) using the standard three electrode cell system with a modified glassy carbon electrode (GCE) as the working electrode, an Ag/AgCl (saturated KCl) reference electrode and a platinum wire as the counter electrode. The electrochemical performances of the modified GCE for glucose detetion were assessed by cyclic voltammetry (CV), and amperometry (*I-t*), and electrochemical impedance spectroscopy (EIS) techniques.

### Preparation of NiWO_4_-modified electrodes

The modified GCEs were prepared by first dispersing *ca*. 5 mg of the as-synthesized NiWO_4_ powder in ethanol solution under sonication treatment for 2 h. Subsequently, *ca*. 8 μL of the dispersed solution was drop casted on the well pre-cleaned surface of the GCE, followed by drying in an oven at room temperature. Prior to each electrochemical measurement, the modified GCE was rinsed with deionized water to remove the loosely bounded materials.

## Additional Information

**How to cite this article**: Mani, S. *et al*. Hydrothermal synthesis of NiWO_4_ crystals for high performance non-enzymatic glucose biosensors. *Sci. Rep*. **6**, 24128; doi: 10.1038/srep24128 (2016).

## Supplementary Material

Supplementary Information

## Figures and Tables

**Figure 1:**
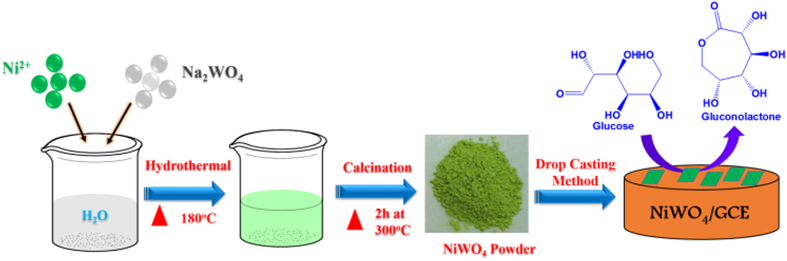
The synthesis route for NiWO_4_ material and its application as high-performance non-enzymatic glucose sensors.
