# Supplementary material for: Hydrothermal synthesis of NiWO4 crystals for high performance non-enzymatic glucose biosensors
Source: Sci Rep. 2016 Apr 18;6:24128. doi: 10.1038/srep24128 (PMC4834566; doi:10.1038/srep24128)
Supplement: Supplementary Information [file srep24128-s1.docx]

**Supporting Information**

**Hydrothermal synthesis of NiWO_4_ crystals for high performance non-enzymatic glucose biosensors**

Sivakumar Mani^1^, Veeramani Vediyappan^1^, Shen-Ming Chen^1^, Rajesh Madhu^1^, Veerakumar Pitchaimani^2^, Jia-Yaw Chang^3^ & Shang-Bin Liu^2,4^

^1^Department of Chemical Engineering and Biotechnology, National Taipei University of Technology, Taipei 10608, Taiwan. ^2^Institute of Atomic and Molecular Sciences, Academia Sinica, Taipei 10617, Taiwan.^3^Department of Chemical Engineering, National Taiwan University of Science and Technology, Taiwan. ^4^Department of Chemistry, National Taiwan Normal University, Taipei 11677, Taiwan.

Correspondence and requests for materials should be addressed to S.M.C. and S.B.L. ([smchen78@ms15.hinet.net](mailto:smchen78@ms15.hinet.net) &[sbliu@sinica.edu.tw](mailto:sbliu@sinica.edu.tw)).


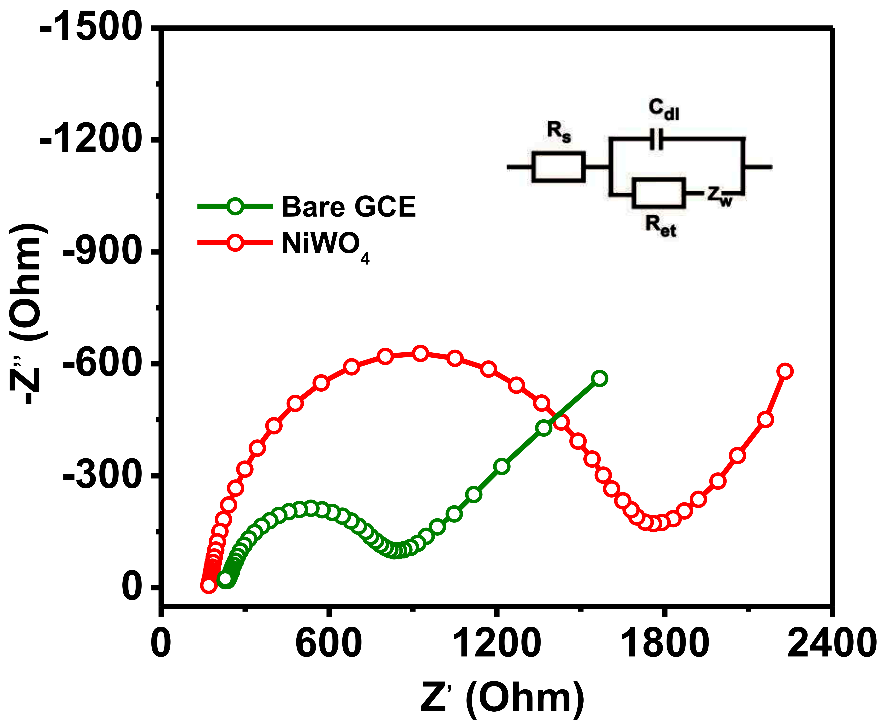


**Figure S1.** Nquist plot for NiWO_4_/ GCE and bare GCE in the presence of 0.1 M KCl solution containing 5.0 mM [Fe(CN)6]^3−/4^ electrolyte.

Table S1. Comparisons of analytical parameters for non-enzymatic glucose sensor over various modified GCEs.

| **Modified electrode** | **Linear range**  **(µM)** | **Detection limit**  **(µM)** | **Sensitivity**  **(µA mM^−1^ cm^−2^)** | **Detection method** | **Ref.** |
| --- | --- | --- | --- | --- | --- |
|  |  |  |  |  |  |
| NiO nanoflakes | 10-800 | 1.2 | 8500 | Amperometry | 1 |
| Ni–MWCNT | 3.2–17.5 | 0.89 | 67.2 | Amperometry | 2 |
| Ni(OH)_2_/rGO | 2–3100 | 0.60 | 11.43 | Amperometry | 3 |
| NiO | 0–8000 | 1.0 | 123 | Amperometry | 4 |
| ZnO-NiO | 0.5–6400 | 0.5 | 120.5 | Amperometry | 5 |
| NiO-Au | Upto 4550 | 1.32 | 48.35 | Amperometry | 6 |
| NiO | 2–10 μM,  0.05–3.3 mM | 0.3 | - | Amperometry | 7 |
| NiO-Ag nanofiber | 1–590 | 1.37 | 19.30 | Amperometry | 8 |
| NiO-Cu | 0.5-5000 | 0.5 | 171.8 | Amperometry | 9 |
| NiWO_4_ | 0.006-4122 µM | 0.18 | 269.83 | Amperometry | This work |

**References**

1. Wang, G. *et al.* Free-standing nickel oxide nanoflake arrays: synthesis and application for highly sensitive non-enzymatic glucose sensors. *Nanoscale* **4**, 3123–3127 (2012).
2. Sun, A., Zheng, J. & Sheng, Q. A highly sensitive non-enzymatic glucose sensor based on nickel and multi-walled carbon nanotubes nanohybrid films fabricated by one-step co-electrodeposition in ionic liquids. *Electrochim Acta* **65**, 64–69 (2012).
3. Zhang, Y. *et al.* Assembly of Ni (OH) 2 nanoplates on reduced graphene oxide: a two dimensional nanocomposite for enzyme-free glucose sensing. *J. Mater. Chem.* **21**, 16949–16954 (2011).
4. Ibupoto, Z. H., Khun, K., Beni, V. & Willander, M. Non-Enzymatic Glucose Sensor Based on the Novel Flower Like Morphology of Nickel Oxide. *Soft Nanosci. Lett.* **3**, 46−50 (2013).
5. Liu, Y. *et al.* Mesoporous ZnO-NiO architectures for use in a high-performance nonenzymatic glucose sensor. *Microchim Acta* **181**, 1581–1589 (2014).
6. Dinga, Y., Liua, Y., Parisi, J., Zhang, L. & Lei, Y. A novel NiO–Au hybrid nanobelts based sensor for sensitive and selective glucose detection. *Biosens.Bioelectronics* 28, 393–398 (2011).
7. Liu, S., Yu, B. & Zhang, T. A novel non-enzymatic glucose sensor based on NiO hollow spheres. *Electrochimica Acta* **102**, 104–107 (2013).
8. Ding, Y., Wang, Y., Su, L., Zhang, H. & Lei, Y. Preparation and characterization of NiO–Ag nanofibers, NiO nanofibers, and porous Ag: towards the development of a highly sensitive and selective non-enzymatic glucose sensor. *J. Mater. Chem.* **20**, 9918–9926 (2010).
9. Zhang, X. *et al.* Porous Cu–NiO modified glass carbon electrode enhanced nonenzymatic glucose electrochemical sensors. *Analyst* **136**, 5175–5180 (2011).
